# Development and application of a novel suspension concentrate for seed coating of rice for controlling bakanae disease and seedling rot disease

**DOI:** 10.3389/fbioe.2024.1418313

**Published:** 2024-06-06

**Authors:** Xue-Xiang Ren, Li Chen, Yu-Chi, Zhao-Li, Xian-Yan Su, Shun-He, Zheng-He Ye

**Affiliations:** ^1^ Institute of Protection and Agro-Products Safety, Anhui Academy of Agricultural Science, Hefei, China; ^2^ School of Plant Protection, Anhui Agricultural University, Hefei, China; ^3^ College of Plant Science and Technology, Huazhong Agricultural University, Wuhan, China

**Keywords:** bakanae disease, control effect, Fusarium moniliforme Sheldon, suspension concentrate for seed coating of rice, nonionic surfactants security

## Abstract

The main rice planting areas in the middle and lower reaches of the Yangtze River are primarily affected by two types of rice seedling diseases: bakanae disease and seedling rot disease. These diseases lead to considerable losses. Seed coating technology effectively protects rice from these diseases and mitigates environmental pollution. We determined the antifungal activity of six fungicides, including phenamacril, azoxystrobin, fludioxonil, metconazole, thifluzamide and prothioconazole against *Fusarium moniliforme* Sheldon and *Curvularia lunata* in this study. In addition, the impact of fungicides and surfactants on rice seed germination were determined. Furthermore, phenamacril and fludioxonil were selected as the active components of suspension concentrate for seed coating. The antifungal activity of phenamacril against *F*. *moniliforme* Sheldon was 0.139 mg/L and fludioxonil against *C*. *lunata* was 0.110 mg/L. PEG-2000 was selected as the surfactant due to its promoting effect on rice seedling. Based on the above findings, 6% phenamacril fludioxonil FS was developed via the wet sand grinding method. The toxicity of 6% phenamacril fludioxonil FS to zebrafish was verified, and field experiments were conducted in five different regions of the Yangtze River Basin. The results indicated minimal toxicity of 6% phenamacril fludioxonil FS to zebrafish. Relative to the control agent consisting of 6.25% phenamacril metalaxyl-M FS, 6% phenamacril fludioxonil FS showed better control effect and exhibited superior efficacy in promoting growth and increasing yield in all five regions. Specifically, the control effect of 6% phenamacril fludioxonil FS on bakanae exceeded 84.83% with the highest yield increasing value recorded at 30.48%. Currently, the market offers a limited selection of suspension concentrate for seed coating of rice. The findings of this study may offer a viable alternative formulation and directions for further research concerning the application of suspension concentrate for seed coating of rice.

## 1 Introduction

Rice (*Oryza sativar* L.) is one of the most important commercial crops and serves as the staple energy source for more than half the global population. In addition, it is also the most widely grown and consumed staple food in Asian countries, thereby playing an important role in global food security (Fukagawa and Ziska, 2019). There are various diseases in rice seeds and seedlings stage during germination and growth, which poses a major risk to global rice security (Imolehin, 1983). Among the numerous diseases affecting rice, seed-borne diseases such as bakanae and seedling rot disease are particularly important fungal diseases (An et al., 2023). Bakanae, commonly known as foolish seedling, poses a considerable threat to rice yield in various rice planting countries worldwide (An et al., 2023). Research has demonstrated that bakanae disease is caused by sever seed-borne *Fusarium* species, with *Fusarium fujikuroi,* which is a member of the *Gibberella fujikuroi* species complex, being the main contributor (Amoah et al., 1995; Desjardins et al., 2000; Wulff et al., 2010). Seedling rot disease is caused by various seed-borne and soil-borne fungal pathogens, including *Curvularia lunata*, *Fusarium oxysporum*, and *Rhizoctonia solani* (Verma et al., 2018; Song et al., 2021; Liu et al., 2022). Seedling rot disease influences the seeds and seedlings of rice plants, with discernible disease symptoms typically emerging shortly after seed sowing (Limtong et al., 2020). Bakanae and seedling rot diseases have result in considerable reduction in seedling production (Ito and Tokunaga, 1933).

The primary approach for managing rice bakanae and seedling rot disease, caused by fungi, involves the utilization of seed soaking and the application of liquid-based seed coating agents (Zeng and Wang, 2010; Adachi et al., 2012). Seed-coating techniques are currently utilized across agricultural regions worldwide (Zhang et al., 2011; Gorim and Asch, 2015). Studies have substantiated that this agricultural technology has a beneficial effect in preventing plant diseases, improving the seed germination rate, promoting shoot growth, and increasing both crop yield and quality (Richard, 2005; Wang et al., 2016; Mnasri et al., 2017; Ren et al., 2019). Despite certain advancements, previous research on seed coatings has primarily focused on the use of toxic fungicides, such as carbofuran, which retains pesticide residue, poses risks to human and livestock health, and causes environmental pollution; furthermore, the outcomes are not consistently satisfactory (Zhu, 2007; Zeng et al., 2018). The performance requirements for suspension concentrate for seed coating of rice are more stringent due to the prolonged (up to 1–2 months) soaking in water required for rice cultivation (Mnasri et al., 2017). At present, most suspension concentrate for seed coating of rice on the market fail to meet the necessary soaking requirements. Consequently, the demand for an innovative, environmentally friendly seed-coating agent as an alternative to conventional coatings is imperative in both agricultural and environmental conservation.

In this study, our aim was to evaluate the antifungal activity of six fungicides, including phenamacril, against bakanae disease and seedling rot disease. A novel eco-friendly rice coating agent, composed of phenamacril and fludioxonil (6% phenamacril fludioxonil; PF), was successfully developed. These active ingredients serve as substitutes for the toxic active ingredients found in traditional toxic products. Subsequently, the efficacy of PF was assessed both in laboratory and field experiments were conducted to assess compared to the conventional suspension concentrate for seed coating (6.25% phenamacril metalaxyl-M; PM) in controlling bakanae in rice plants. In addition, the impacts of fungicides on seed germination and growth were also examined. Lastly, in order to verify the environmental safety of PF, the biosafety of PF was investigated using zebrafish as the study subject. This study offers a novel seed-coating agent that can effectively control rice seedling diseases and reduce labor costs. It also has specific guiding significance for theoretical research and holds promising potential for application in rice production.

## 2 Materials and methods

### 2.1 Materials and reagents

High-purity pesticide standards of phenamacril (99.7% purity), fludioxonil (99.8% purity), azoxystrobin (99.9% purity), metconazole (99.5% purity), thifluzamide (99.6% purity), and prothioconazole (99.2% purity) were acquired from Suolaibao Laboratory Technologies Inc. (Beijing, China). The emulsifiers (LAE-9, S-20, SG-6, A-115, BY-140, and EL-60), ethylene glycol, polyethylene glycol 2000 (PEG-2000), and other chemicals of analytical reagent grade were obtained from Sinopharm Chemical Reagent Company (Shanghai, China). The red pigment, potato dextrose agar (PDA), and hybrid rice seed (Shenliangyou 8010) were purchased from Shandong Jinnuo Co., Ltd. (China). Zebrafish (*Danio rerio*) weighing 0.4 ± 0.1 g were obtained from the China Zebrafish Resource Center. The traditional rice seed-coating agent, 6.25% phenamacril · metalaxyl-M (PM), was purchased from Zhengzhou Seed Technology Developed Limited Company in Henan, China. Deionized water was utilized for all of the tests, with the exception of the biosafety test.

### 2.2 Fungal pathogens

The fungal pathogens *C. lunata* DOAC 2313 and *F*. *moniliforme* Sheldon, responsible for rice bakanae and seedling rot disease, were isolated from infected rice plants in Qianshan (China). The fungal strains were cultured in PDA at 25°C in the dark and subsequently stored at 4°C.

### 2.3 *In vitro* evaluation of antagonistic activities of fungicides against fungal pathogens causing bakanae and seedling rot disease in rice plants

The antifungal activity of fungicides to *C. lunata* DOAC 2313 and *F. moniliforme* Sheldon was tested using the hyphal growth method (Mnasri et al., 2017). Add pesticide into PDA medium, and then the mixtures were poured into dishes. In brief, a 2.5-mm mycelial plug was transferred from the margin of 3-day-old colony to the center of PDA plate amending with different fungicide concentrations, and then cultured at 25°C for 3 days. A PDA dish inoculated only with the fungal pathogen was designated as the control. Each treatment was performed in triplicate. The diameter of the colony (mm) on the PDA was measured using a cross-measuring method, and the final result was the average value of the three replicates. The percentage of fungal pathogen growth inhibition was calculated by the formula (Wang et al., 2016): Growth inhibition (%) = (Diameter of control-Diameter of treatment)/(Diameter of control–0.6 cm) × 100.

### 2.4 Preparation of a novel suspension concentrate for seed coating

The novel suspension concentrate for seed coating of rice was prepared using the wet sand processing superfine grinding method (Gálvez et al., 2019). Through an orthogonal test, we determined the optimal formula for the PF suspension concentrate for seed coating. The novel coating (i.e., PF) was prepared under the following procedural conditions: specific amounts of phenamacril, fludioxonil, LAE-9, S-20, SG-6, A-115, BY-140, EL-60, magnesium aluminum silicate, polyethylene glycol, and red pigment were mixed separately and gradually introduced into an aqueous solution in accordance with the desired based on certain ratios. Subsequently, the mixture was stirred at 25°C under normal pressure for 2–3 h until completely dissolved. Additional additives, including the film-forming auxiliaries, were incorporated into the aqueous solution according to predetermined ratios. With this, the development of a novel suspension concentrate for seed coating of rice was finalized. Meanwhile, the stability was assessed using the recommended Collaborative International Pesticide Analytical Council (CIPAC) method, which fully met the requirements for pesticide preparation.

### 2.5 Laboratory method for the germination test

Rice seeds for examination were coated with PF via stirring, and the coating agent was applied at specific coating ratios. Following the application of the coating, the seeds were air-dried for 20 min at 25°C–35°C. To prepare them for use, all soaking treatments were carried out at 30°C for 24 h. Seven distinct coating treatments were employed: 1) phenamacril coating; 2) fludioxonil coating; 3) azoxystrobin coating; 4) metconazole coating; 5) thifluzamid coating; 6) prothioconazole coating; and 7) uncoated rice seeds (CK) as a contrasting control. The rules for seed testing of all treatments were determined by the International Seed Testing Association (ISTA) (Liang et al., 2021). Samples of 300 seeds of each treatment were arranged on two layers of wet filter paper in three Petri dishes (100 seeds per dish) with three replications. The seeds were placed in a constant temperature and humidity incubator (28°C, a photoperiod of 12 h, and 80%–85% relative humidity). Germination assessments were performed daily, starting on the seventh day. After 14 days of incubation, 15 seedlings were randomly selected from each treatment to evaluate the seedling quality. Germination was calculated using the following formula:

$$\text{Germinability }\left(\%\right)=\text{GS}7\;/\text{ TS}\times 100\%$$

GS7 represents the number of germinated seeds on the seventh day; TS denotes the number of total seeds investigated.

### 2.6 Biosafety evaluation of PF in zebrafish

We conducted a biosafety assessment to estimate whether PF posed risk the public during its production, use, and disposal. Ten zebrafish were placed in separate plastic containers (cuboid shape, 13.4 cm × 7.8 cm × 6.5 cm) containing PF aqueous suspensions (300 mL) of varying concentrations (0, 30, 61.25, 125, 250, and 500 μg/mL). After the final feeding, the number of deceased fish was calculated at 12-h intervals under still-water conditions. After 10 days, the viability of the zebrafish was recorded by counting the number of surviving zebrafish.

### 2.7 Field trial

The superior formulation was selected based on laboratory tests, optimized for parameters (e.g., pH) and evaluated in the field. Field trials were conducted in 2020–2022 in the experimental fields in Anhui (Qian shan), Hubei (Qian jiang and Wu han), Hunan (Tao yuan), and Jiangsu (Yang zhou), China, utilizing PF and PM. All rice seeds were manually film-coated and the seed-coating agents were mixed in a ratio of 1:150. Following the application of the coating, the seeds were subjected to air drying for 30 min at 25°C–35°C. Subsequently, all soaking treatments were conducted at 30°C for 24 h. The treated and uncoated seeds were sown in randomly arranged plots in triplicate. Each plot of 20 m^2^ (consisting of 10.0 m × 2.0 m) had six rows with a spacing of 60 cm. A total of 250 rice seeds were planted on every plot. A random sample of 100 seedlings was chosen to measure root and shoot lengths and fresh and dry weights, before transplanting from each plot. Subsequently, an additional 100 plants were randomly selected from each plot to assess the control effect on rice bakanae disease. Uniform field management was implemented across all experimental plots.

### 2.8 Statistical analysis

The data were presented as the mean ± standard deviation. The data from each experiment were statistically analyzed using one-way analysis of variance (ANOVA), followed by Tukey’s multiple-range test as available on the SPSS 22.0 statistical package (ver. 22.0; SPSS Company, Chicago, United States) (Liang et al., 2021). The data were analyzed at significance level of *p* < 0.05. In the zebrafish biosafety experiment, the antifungal activity regression equation and LD50 values were obtained via adaptive probability analysis. Additionally, the laboratory experiments and field trials were calculated and subjected to statistical analysis. Figures were generated using GraphPad Prism 7 (GraphPad Software, Inc., United States).

## 3 Results

### 3.1 Antifungal activity of fungicides against *F*. *moniliforme* Sheldon and *C. lunata*

Table 1 displays the antifungal activity of various fungicides against *F*. *monili-forme* Sheldon and *C. lunata*. In this study, significant toxicity differences were observed between various fungicides against *F*. *moniliforme* Sheldon and *C. lunata*. Metconazole showed significant antifungal activity in the control of *F*. *moniliforme* Sheldon with an EC_50_ (half-maximal effective concentration) of 0.015 mg L^−1^ after 3 days (Table 1). Similarly, fludioxonil demonstrated evident inhibitory activity in controlling *C. lunata* (EC_50_ = 0.110 mg L^−1^). In addition, we discovered that phenamacril treatment exhibited inhibitory effects against *F*. *moniliforme* Sheldon (EC_50_ = 0.139 mg L^−1^) and *C. lunata* (EC_50_ = 0.231 mg L^−1^).

**TABLE 1:** Effectiveness of fungicides against *F*. *moniliforme* Sheldon and *C*. *lunata*.

Fungicide	*F*. *moniliforme* Sheldon		*C. lunata*	
	EC_50_ (mg L^−1^)	95% FL (mg L^−1^)	EC_50_ (mg L^−1^)	95% FL (mg L^−1^)
Phenamacril	0.139	0.054–0.317	0.231	0.103–0.356
Azoxystrobin	3.069	1.271–5.366	7.735	3.241–10.366
Fludioxonil	7.472	3.351–10.669	0.110	0.023–0.217
Metconazole	0.015	0.006–0.041	0.647	0.244–0.910
Thifluzamide	10.545	6.106–15.313	5.741	3.271–8.363
Prothioconazole	2.279	1.017–3.662	29.270	13.671–55.306

EC_50_, the half-maximal effective concentrations; FL, fiducial limit.

In general, compounding application of pesticides with various mechanisms was an efficient strategy for inhibiting the growth of plant diseases, thus reducing the amount of chemical pesticides required (Standardization Administration of the People’s Republic of China, 2014); therefore, the utilization of synergistic combinations of two fungicides on *F*. *moniliforme* Sheldon and *C. lunata* was examined. The results indicated that compound fungicides at the ratio 1:1 had a significant synergistic effect against *F*. *moniliforme* Sheldon and *C*. *lunata*, the CTC values were 1.07 and 0.98, respectively.

### 3.2 Effect of fungicides and nonionic surfactants on seed germination and seedling growth

**FIGURE 1:**
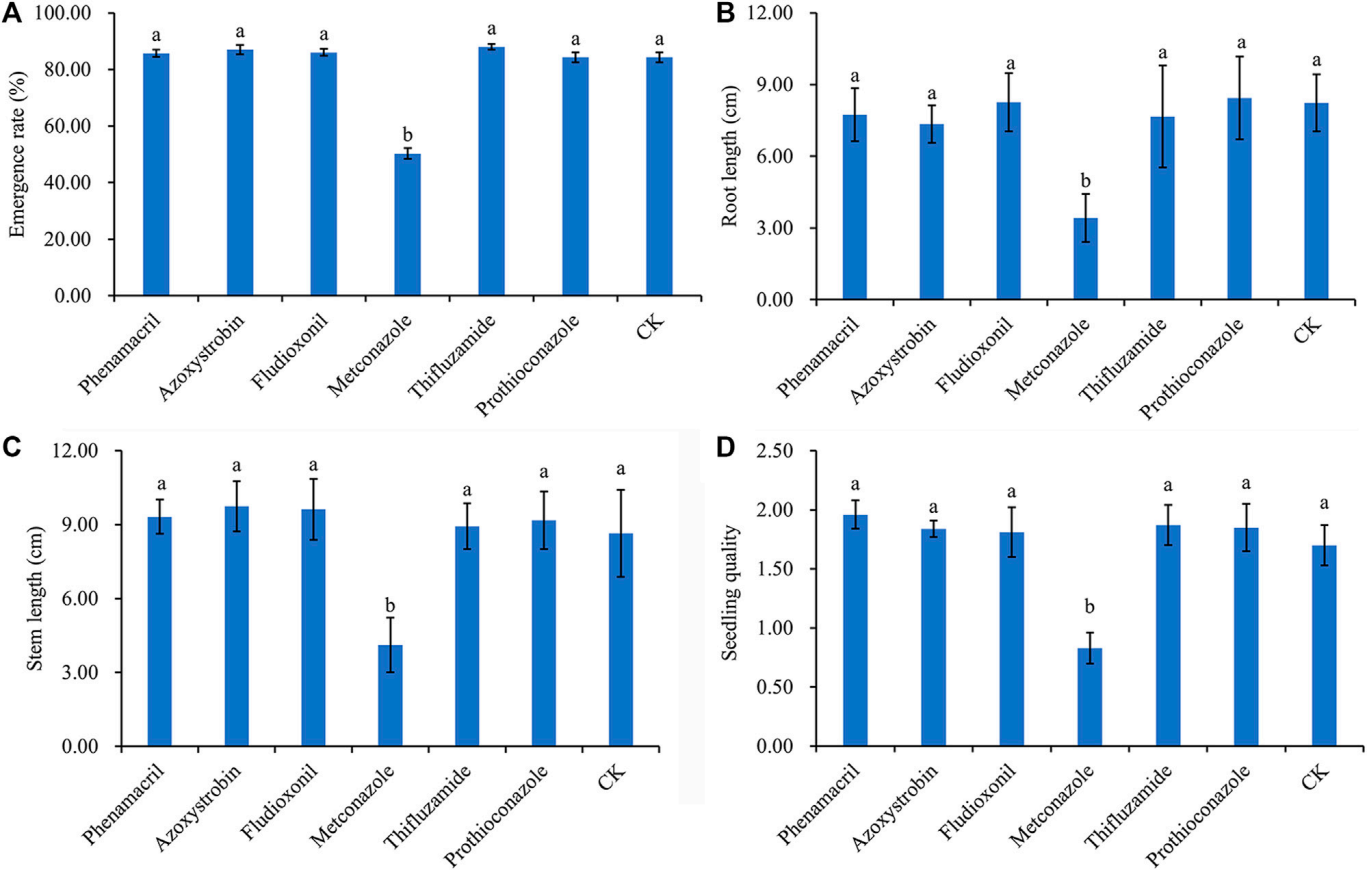
Effects of different fungicides on germination **(A)**, root length **(B)** and stem length **(C)**, and dry weight **(D)** of rice.

The effects of different fungicides and nonionic surfactants on rice seed germination and seedling quality were investigated in the laboratory (Figure 1; Table 2). The effects of the fungicides on rice germination and growth were depicted in Figure 1. All fungicides had a positive effect on germination rate, stem and root length, and dry weight except for metconazole, which had obvious negative effects, and its inhibition of germination rate, stem length, and dry weight were statistically significant (*p* < 0.05). As shown in Figure 1, there was no significant difference between the other fungicides and CK. Phenamacril increased the dry weight of rice plants, whereas fludioxonil increased the root and stem length.

**TABLE 2:** The effects of various surfactants on seedling quality.

Surfactants	Reagent concentration (g/L)	Seedling quality			
		Emergence rate (%)	Root length (cm)	Stem length (cm)	Dry weight (g)
LAE-9	0.0002	82.3 a	8.17 a	7.79 a	1.73 a
S-20	0.0002	78.0 b	7.26 a	7.87 a	1.61 b
SG-6	0.0002	75.7 b	7.71 a	8.03 a	1.68 a
A-115	0.0002	80.0 a	7.63 a	7.93 a	1.72 a
BY140	0.0002	87.3 a	8.01 a	7.89 a	1.65 ab
EL-60	0.0002	82.0 a	7.32 a	8.24 a	1.67 a
JFC-E	0.0002	76.0 b	7.56 a	7.73 a	1.71 a
PEG-2000	0.0002	85.0 a	8.64 a	8.71 a	1.78 a
CK	—	84.3 a	8.23 a	8.64 a	1.70 a

This table presents the mean and the standard error of three replicates to compare the differences between seven treatments simultaneously. Different lowercase letters indicate significant differences in each column (*p* < 0.05).

Based on the results of the antifungal activity and fungicide safety experiments, it is recommended to use phenamacril and fludioxonil as the active constituents in the coating formulation. The mass ratio of phenamacril to fludioxonil was 1:1, and bakanae disease and seedling rot disease could be effectively controlled at this ratio.

We also evaluated the effects of different nonionic surfactants on rice seedling quality. Surfactants also form important components of seed-coating agents, improving distribution across the seed surface, usually between 3% and 12% of the formulation by weight (Gesch and Archer, 2005; Magalhaes et al., 2009; Chen et al., 2016; Saeed et al., 2016). The results showed that eight frequently used nonionic surfactants had different effects on the germination of rice seeds (Table 2). By comparison, S-20, SG-6, and JFC-E induced the largest negative effects, which could significantly reduce the emergence rate. This was followed by JFC-E, A-115, EL-60, and LAE-9. There were no differences with CK in the inhibition of weight and germination rate (*p* < 0.05). In contrast, PEG-2000 increased the germination rate (85.0%), root and stem length (8.64 cm and 8.71 cm, respectively), and dry weight (1.78 g) of rice seedlings. And there was no significant difference between PEG-2000 and CK. Therefore, PEG-2000 was identified as a safe nonionic surfactant because of its positive effects on rice seedlings.

### 3.3 Determination of suspension concentrate for seed coating

The antifungal activity, germination percentage, and seedling quality of different fungicides and nonionic surfactants were evaluated and analyzed to determine the optimal formula for laboratory experiments. Surfactants and film-formers for the seed-coating agent were assessed using an orthogonal test. Based on these indicators, an optimal formula for the resulting suspension concentrate for seed coating was obtained. The detailed formulations are presented in Table 3. Based on the above optimal formula, a suspension concentrate for seed coating of rice was prepared, and if the main performance indexes met the agent requirements, this modified formula was used for the next step of the field trial.

**TABLE 3:** Main components of the novel suspension concentrate for seed coating (PF).

Component	Content % (g/g)	Property
Phenamacril	3.00	Active ingredient
Fludioxonil	3.00	Active ingredient
Sodium ligninsulfonate	0.80	Nonionic surfactant
PEG-2000	4.00	Wetting dispersant
Polyacrylamide carboxymethyl cellulose	3.00	Film-forming agent
Ethylene glycol	4.00	Anti-foaming agent
Xanthan gum	0.15	Thickener
Pigment red	6.00	Dye agent
Deionized water	76.05	—

The suspension concentrate for seed coating was developed using the wet sand grinding method. In this formula, we used sodium ligninsulfonate as a dispersant, which acted as a steric hindrance, and when the amount of PEG-2000 reached 4%, it had a good wetting effect. When polyacrylamide carboxymethyl cellulose and xanthan gum were used as filmogens, they could be soaked in water for 15 days without falling off, demonstrating excellent performance as filmogens for rice seed coatings. Ethylene glycol was used as an antifreezer, and when the temperature was below zero, the fluidity of the formula was guaranteed. We used red pigment as a warning color because red is more eye-catching than other colors, such as blue.

### 3.4 Biosafety evaluation of suspension concentrate for seed coating

**FIGURE 2:**
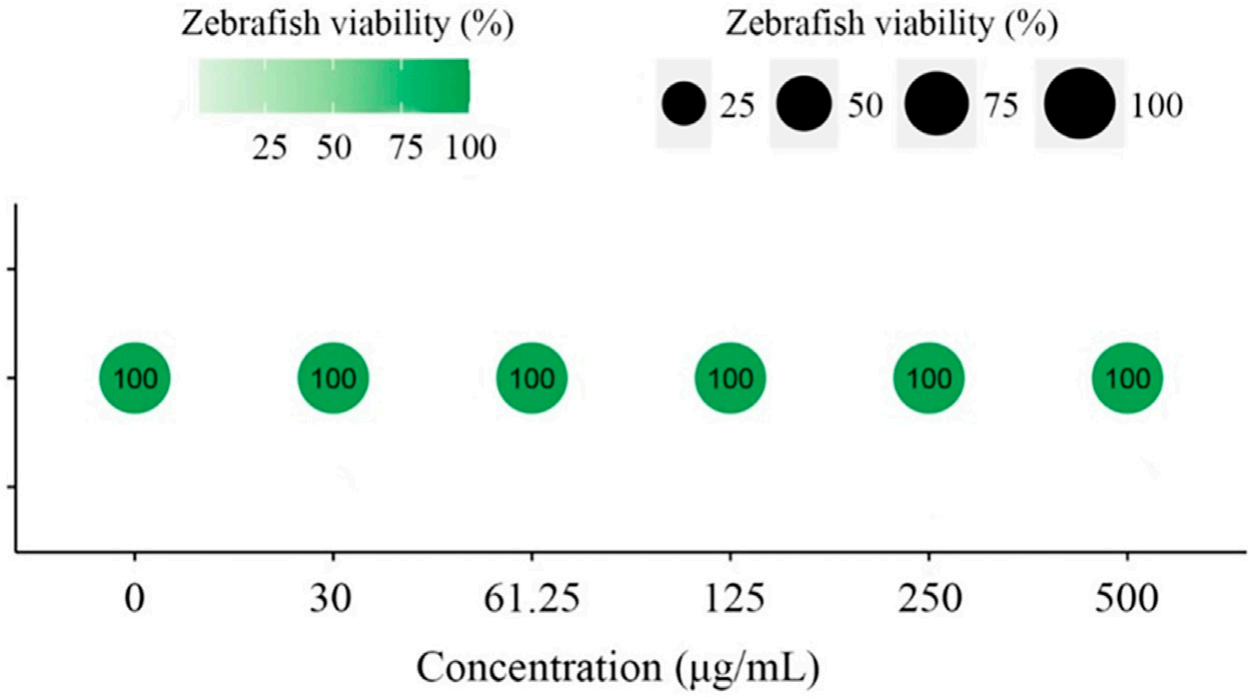
Viabilities of zebrafish in aqueous solutions of different concentrations of PF seed-coating agents. Standard deviations (*n* = 3) are represented by error bars.

The effect of PF on zebrafish was investigated to evaluate the biosafety of PF seed-coating. As illustrated in Figure 2, different PF concentration treatments (0, 30, 61.25, 125, 250, and 500 μg/mL) all exhibited high levels of surviving zebrafish and displayed no significant influence on zebrafish after 96 h. This result indicated that PF has a high safety index for fish according to domestic standards. Therefore, the examined PF seed-coating agent possessed good biosafety from an environmental protection perspective.

### 3.5 Experimental results of suspension concentrate for seed coating in the field trial

**FIGURE 3:**
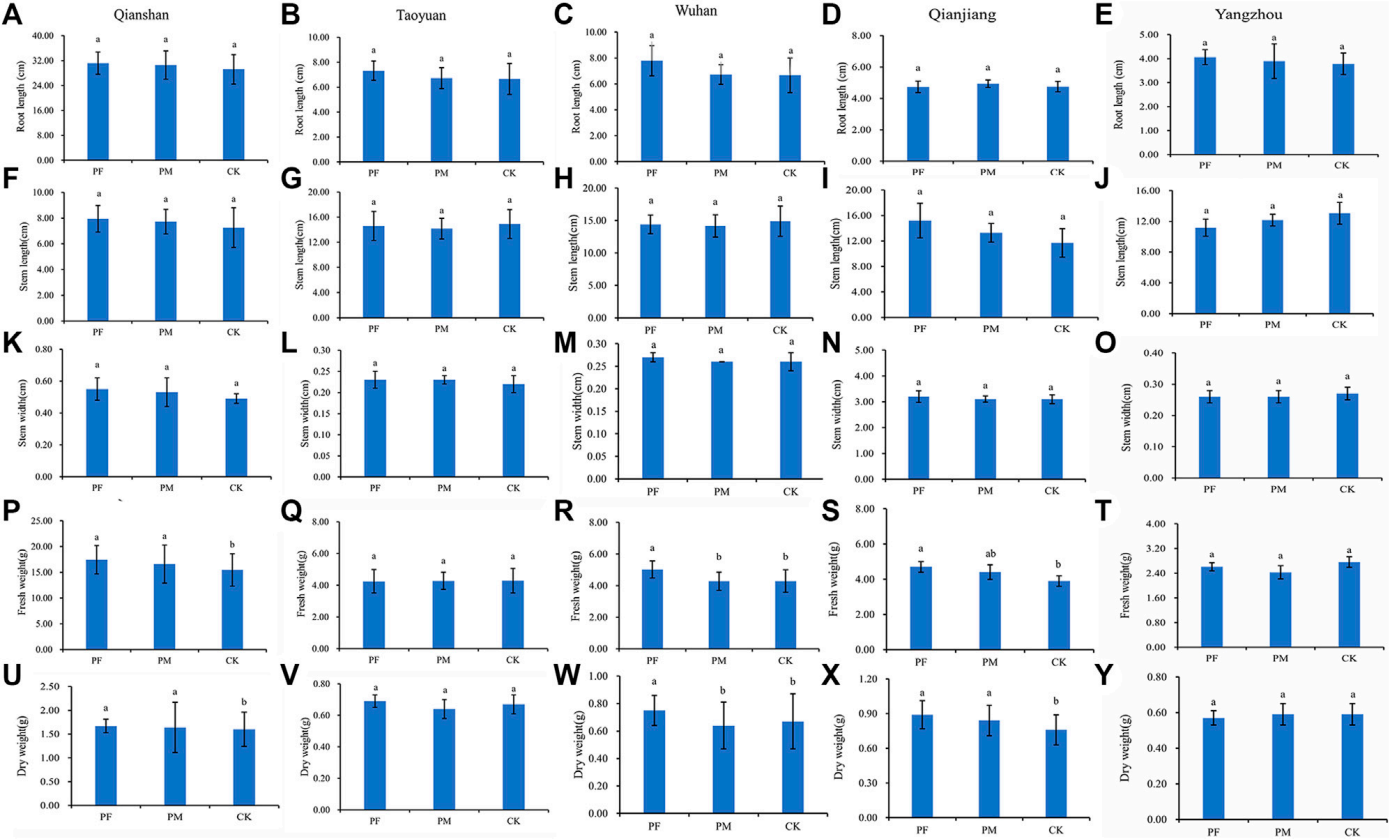
Effects of seed coating treatments on the root length (A-E), stem length (F-J), stem width (K-O), fresh weight (P-T), and dry weight (U-Y) of rice in the field trial. CK, uncoated seeds; PF, PF-coated seeds; PM, PM-coated seeds. This figure presents the mean and standard error of three replicates to compare the differences between seven treatments simultaneously. Distinct lowercase letters in the same column indicate significant differences (*p* < 0.05).

**FIGURE 4:**
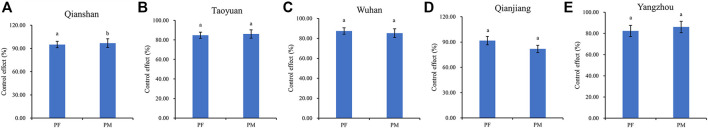
Effects of seed coating treatments on rice in the field trial. **(A)** Qianshan; **(B)** Taoyuan; **(C)** Wuhan; **(D)** Qianjiang; **(E)** Yangzhou. CK, uncoated seeds; PF, PF-coated seeds; PM, PM-coated seeds. This figure presents the mean and standard error of three replicates to compare the differences between seven treatments simultaneously.

**FIGURE 5:**
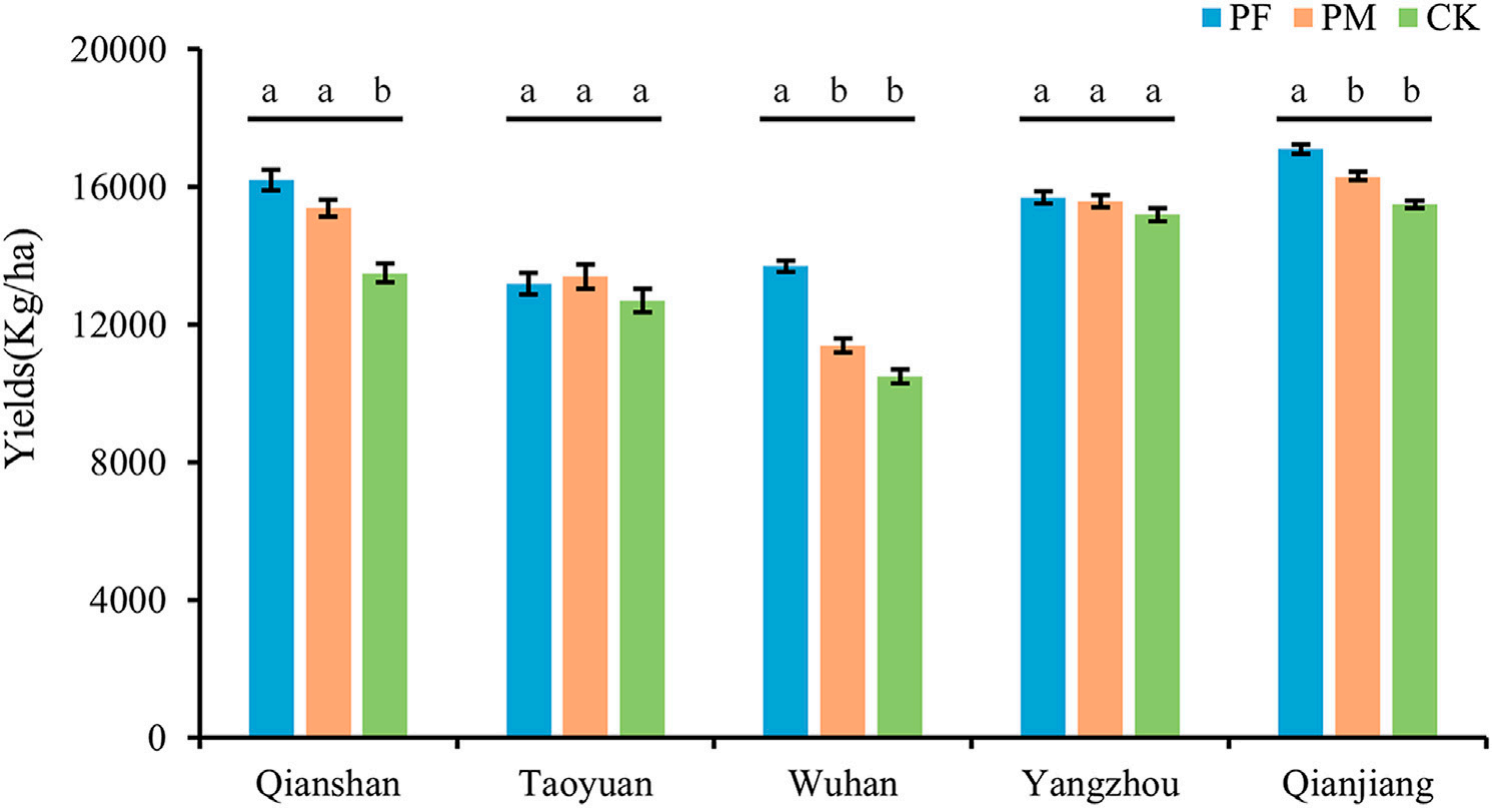
Rice yields in five different regions. This figure presents the average of three replicates. Distinct lowercase letters in the same column indicate significant differences (*p* < 0.05).

Field trials of PF, PM, and uncoated samples were conducted in the experimental plots, with the results depicted in Figures 3–5. Notably, PF with a 1:150 ratio clearly demonstrated superior results relative to PM and CK across all key performance indicators (Figure 3). In Qianshan, fresh weights ranged from 15.46 to 17.43 g, with dry weights ranging from 1.60 to 1.67 g (Figures 3P, U). In Taoyuan, fresh weights varied from 4.25 to 4.29 g, with dry weights ranging from 0.64 to 0.69 g (Figures Q, V). Moving to Wuhan, fresh weights ranged from 4.28 to 5.02 g, with dry weights varying from 0.64 to 0.75 g (Figures 3R, W). In Qianjiang, fresh and dry weights spanned from 3.90 to 4.70 g and 0.76–0.89 g (Figures 3S, X), respectively. Fresh weights from 2.43 to 2.76 g with dry weights ranging from 0.57 to 0.59 g in Yangzhou (Figures 3T, Y). Compared to PM, a traditional suspension concentrate for seed coating of rice, PF had a significant effect on the biomass, stem length (Figures 3F–J), and root length (Figures 3A–E) of rice in the field trial. Meanwhile, PF was best at promoting the germination and growth of rice in Qianshan and Wuhan, respectively, resulting in higher rice biomass compared to the positive control, PM. There were no difference of stem width between the different areas (Figures 3K–O).

The field trial also included an evaluation of how well the suspension concentrate for seed coating controlled soil-brone diseases (Figure 4). In this study, we investigated bakanae because it was the only disease that occurred in the field during the year. The results showed that PF and PM effectively controlled and reduced disease severity at five field sites, and the control efficiency reached 94.95% and 96.81%, respectively. Notably, in the Qianshan field, PF demonstrated the highest control efficiency at 94.95%, followed by PF in the Qianjiang field at 91.67%.

The results in Figure 5 showed that in five different regions of the Yangtze River Basin, the application of suspension concentrate for seed coating can effectively increase rice yield. This was the most significant difference in Qianshan, Qianjiang, and Wuhan between the CK and PF and PM treatments. However, there were no significant differences between PF and PM in the five regions, although PF could have increased production.

These results indicated that utilizing PF as the suspension concentrate for seed coating effectively controls bakanae and has a positive effect on the germination, growth, and yield of rice in the field. The correlation analysis demonstrated a significant positive correlation between the main performance indexes observed in the field trial and those obtained in the laboratory experiment. In summary, the use of the PF formulation has effectively controlled foot and root diseases in irrigated rice, resulting in enhanced yield in the Yangtze River Basin. Therefore, the introduction and application of suspension concentrate for seed coating in agriculture are useful.

## 4 Discussion

Seed coating is a kind of seed treatment technology and has been successfully applied in modern agriculture. It can be seen from China Pesticide Information Network Suspension concentrate for seed coating is the most commonly used dosage form of seed coating. The film forming agent contained in the suspension concentrate for seed coating can tightly wrap the active ingredients such as pesticides around the seeds, so that the role of the agent can be maximized. Seed coatings have the ability to effectively reduce the use of pesticides and are becoming more widely used. The application of rice suspension concentrate for seed coating is less prevalent compared to suspension concentrate for seed coating of corn and wheat because of underdeveloped technology (Mnasri et al., 2017). Bakanae is an important disease of rice worldwide and can be spread by seed and soil-borne, with crop yield losses ranging from 3.0% to 95.4% depending on planting areas and variety (Wulff et al., 2010; An et al., 2023). Seed coating represents one of the most effective and straightforward method to control bakanae and seedling rot disease in rice plants (Limtong et al., 2020). Phenamacril is a new cyanoacrylate chemical fungicide that restricts the class I myosin ATPase activity and commonly employed as a seed-soaking agent in China (Hou et al., 2018; Li et al., 2018). However, its efficacy in controlling bakanae and other diseases has declined in recent years (Hou et al., 2018; Wu et al., 2020). Fludioxonil is a type of contact protection fungicide, which has a good preventive effect on a variety of crop, fruit, vegetable diseases, such as wheat scab, strawberry gray mold, cucumber gray mold, potato dry rot (Tiwari et al., 2020; Wang et al., 2022; Wen et al., 2022; Ren et al., 2024)Our experimental results also proved this point. This provides scientific support for prolonging the service life of phenamacril and fludioxonil in the process of rice disease control.

Many pesticides have been shown to be embryonically toxic and teratogenic to non-target aquatic organisms such as fish, amphibians and invertebrates (Pašková et al., 2011). The toxicity of pesticides to non-target aquatic organisms is the key item of pesticide ecological risk assessment (He et al., 2019). For instance, fipronil has been banned from use in rice fields due to its high toxicity to aquatic animals such as fish (Tingle et al., 2003). Zebrafish (*Danio rerio*) constitute the most common model test species, and it has been used in many pesticide risk assessments. In this study, we successfully prepared an eco-friendly suspension concentrate for seed coating of rice composed of phenamacril, fludioxonil, and additional additives (i.e., PF) as an alternative to replace conventional toxic products. The survival rate of zebrafish treated with different concentrations of seed coating agent was higher, and the effect on zebrafish was not significant after 96 h. It showed that the seed coating agent was friendly to aquatic organisms. In addition, seed coating agent is beneficial to the germination of crop seeds and promote growth (Zhang et al., 2022). For instance, plant height, root length, fresh weight and dry weight of cotton seedlings were significantly increased after application of seed coating agent (Tu et al., 2016). In our study, the analysis results from laboratory and field experiments showed that this seed-coating agent could increase seed germination and growth. Moreover, seed coating agent can not only promote plant growth, but also effectively reduce the harm of pests and diseases (Ehsanfar and Modarres-Sanavy, 2005). Our results also showed that the novel uspension concentrate for seed coating could effectively control the incidence of bakanae in rice plants by inhibiting fungal growth. Similar to our results, the risk of wheat scab could be reduced by seeds coating with *Streptomyces* sp. strain DEF39 spores (Mattei et al., 2022). Importantly, PF exhibited a high level of biosafety. Compared to an existing suspension concentrate for seed coating (i.e., PM), the novel suspension concentrate for seed coating (i.e., PF) demonstrated superior performance in increasing production and reducing operating costs.

At present, there is a limited selection of rice seed-coating agents on the market due to the requirement for rice seed-coating agents to possess superior film-forming properties compared to dry farming suspension concentrate for seed coating (Song et al., 2021). In order to ensure seed germination and seedling growth, the film-forming agent applied to the surface of the seed must be both permeable and breathable. In this formulation, we used a combination of polyacrylamide carboxymethyl cellulose and xanthan gum as a filmogen. This combination exhibits excellent water and immersion resistance, making it highly suitable for use as a seed-coating agent for rice. This represents a new discovery. The red pigment not only has a striking stimulating effect but also does not have an adverse effect on rice germination. This study also confirms the safety of surfactant, ensuring the safety of the developed suspension concentrate for seed coating.

## 5 Conclusion

In this study, we developed a rice seed-coating agent by combining phenamacril with fludioxonil. We discovered that this agent has low toxicity to zebrafish and effectively controls bakanae disease in rice plants within the Yangtze River Basin. Seedling rot disease did not occur in the experimental field, thereby rendering statistical analysis unnecessary. Therefore, this study presented a promising approach for disease prevention, promoting the germination and growth of rice, enhancing rice production, and effectively reducing the cost of suspension concentrate for seed coating compared to conventional suspension concentrate for seed coating. This approach holds great potential for practical application.

The 6% phenamacril fludioxonil FS developed in this study can effectively enhance the quality of rice seedlings and is safe for the aquatic indicator organism, zebrafish. It demonstrated a strong controlling effect and outstanding increase in yield for bakanae-infected crops in five different regions of the experiment. This demonstrates that this coating agent can be used for rice seed coatings and serves as an excellent alternative to conventional suspension concentrate for seed coating of rice.

## Data Availability

The original contributions presented in the study are included in the article/Supplementary material, further inquiries can be directed to the corresponding author.
